# Nephrectomy for Large Multiple Abscess of the Kidney*Reported to the Louisville Clinical Society.

**Published:** 1897-04

**Authors:** William C. Duggan

**Affiliations:** Louisville, Ky., Professor of the Principles and Practice of Surgery and Surgical Pathology in the Louisville Medical College, etc.


### NEPHRECTOMY FOR LARGE MULTIPLE ABSCESS OF THE KIDNEY.*

By WILLIAM C. DUGAN, M.D., LOUISVILLE, KY.,

Professor of the Principles and Practice of Surgery and Surgical Pathology in the Louisville Medical College, etc.

The specimen which I present is the left kidney, removed from a patient twenty-two years of age, who had been under observation for several months. I saw her with Dr. Frank before she was operated on at all. There was a large tumor in the region of the left kidney which Dr. Roberts incised and found to be a pus sac. His diagnosis at the time, I understand, was that it was an abscess of the kidney, the urine containing pus. He instituted drainage, and she improved for a while, but soon the abscess refilled and she commenced to do badly. A few weeks ago I was recalled to see the case in consultation with Dr. Kelly. The patient was very thin, and we had no trouble in demonstrating that there was an abscess of the kidney; we could catch the kidney between the hands and by pressing upon it pus was exuded. Dr. Roberts did a nephrotomy, and in the course of the operation opened the upper part of the kidney, but this provided only for the overflow of the

*Reported to the Louisville Clinical Society.

pus. In operating upon the case the usual incision between the last rib and the crest of the ilium was made, and exposed the kidney; then it was separated without any trouble from the surrounding structures, and delivered carefully through the incision. There was one large abscess extending down into the pelvis, another extending well over toward the median line, and another extending up under the liver. The tumor was fully as large as a cocoanut, about twice as long, but after emptying the abscess sacs we had no difficulty in bringing it through the opening and tying off the pedicle. The patient is getting along nicely, and I anticipate no further trouble. The other kidney appears normal and quite able to do double duty—in fact, this specimen has no kidney tissue.

I report the case to emphasize the importance of doing a nephrotomy first, letting the patient come out from under the cloud of sepsis, then doing a secondary nephrectomy afterwards. Had Dr. Roberts attempted this operation, the chances are that he would have lost his case at once. Therefore, in all such cases, do first a nephrotomy, and then as soon as possible the nephrectomy.

